# Lectures on Clinical Medicine

**Published:** 1841-04

**Authors:** 


ART. XIV.—*Leçons de Clinique Médicale faites à l'Hôtel Dieu de Paris.* Par M. le Professeur CHOMEL, recueiliées et publiées par F. SESTIER, M.D., Professeur agrégé à la Faculté de Médecine de Paris, &c. &c. Tome troisième. (PNEUMONIE.)—*Paris*, 1840. 8vo, pp. 592.

*Lectures on Clinical Medicine.* By Professor CHOMEL, reported and published by F. SESTIER, M.D. &c. Third Volume. (PNEUMONIA.)

WHAT claim this volume has to be ushered into the world as a part of M. Chomel's Clinical Lectures we are completely at a loss to divine. The introduction commences by a statement that that Professor has not delivered general lectures upon pneumonia; his occasional observations, therefore, are all the compiler can have availed himself of; and there is positively not an individual of eminence in Paris whose name does not occur more frequently in its pages than that of the clinical teacher of the Hôtel Dieu. We are therefore disposed to think the permission to parade his name on the title-page rather unwarrantably granted by M. Chomel. The work is, in fact, a pure compilation by the professed editor, and a very imperfect one it is. As M. Sestier has understood the office of compilation, this consists in the simple arrangement under proper heads of all the facts and opinions produced by every author within his reach, relieved by an occasional attempt at languid criticism. How the compiler may achieve higher things than this we need not stay to make evident; but even for the humble species of literary drudgery he has chosen to figure in, M. Sestier cannot be said to possess the necessary requisites, for, like the immense majority of his countrymen, his powers as a linguist appear limited to speaking and writing his mother tongue. Notwithstanding all this, we should be sorry to be understood as contesting the utility of this production altogether: as it contains abstracts of the works of the writer's countrymen upon the special characteristics of pneumonia at every period of existence—in new-born infants, in children, in adults, and in subjects of advanced age—it may be referred to with considerable satisfaction by persons unwilling to encounter the labour of perusing the original treatises themselves. With the contents of these treatises we, at the period of their publication, made the readers of this journal very fully acquainted.

Under the head of differential diagnosis we observe the following sufficiently curious remark: "When a very large tumour of the aorta fills a great portion of the thorax, and in consequence of the attenuations of the walls of the sac, a small quantity of its contained blood oozes into the trachea, the dull sound and sanguinolent sputa might lead the observer to admit the existence of pneumonia. A case of this kind was observed in the wards of M. Louis, and another related in one of M. Rostan's works. A woman aged sixty complained of pain at the posterior part of the left side of the chest: the sound, on percussion, was dull, the respiration obstructed, sanguinolent sputa followed coughing, the pulse was frequent and hard, the skin hot, and the patient suffered under considerable thirst. On opening the body the lung was found to be healthy, the aorta the seat of an enormous aneurism." The writer adds, and, so far as we can trust to an *à priori* judgment, we coincide in the opinion, "Nevertheless, ausculation would scarcely allow of any doubt being entertained in a case of this kind; the course and duration of the malady, as well as the antecedent history, would also clear up the diagnosis."

